# Modified Northern blot protocol for easy detection of mRNAs in total RNA using radiolabeled probes

**DOI:** 10.1186/s12864-021-08275-w

**Published:** 2022-01-20

**Authors:** Tao Yang, Mingdi Zhang, Nianhui Zhang

**Affiliations:** Key Laboratory of Bio-Resource and Eco-Environment of Ministry of Education, College of Life Sciences, Sichuan University, Sichuan 610065 Chengdu, People’s Republic of China

**Keywords:** Detection sensitivity, Formaldehyde-agarose gel, Northern blot analysis, Posthybridization wash

## Abstract

**Background:**

Northern blotting is still used as a gold standard for validation of the data obtained from high-throughput whole transcriptome-based methods. However, its disadvantages of lower sensitivity, labor-intensive operation, and higher quality of RNA required limit its utilization in a routine molecular biology laboratory to monitor gene expression at RNA level. Therefore, it is necessary to optimize the traditional Northern protocol to make the technique more applicable for standard use.

**Results:**

In this paper, we report modifications and tips used to improve the traditional Northern protocol for the detection of mRNAs in total RNA. To maximize the retention of specifically bound radiolabeled probes on the blot, posthybridization washes were performed under only with moderate-stringency until the level of radioactivity retained on the filter decreased to 20~50 counts per second, rather than normally under high and low stringency sequentially for scheduled time or under only high stringent condition. Successful detection of the low-expression gene using heterologous DNA probes in 20 µg of total RNA after a two-day exposure suggested an improvement in detection sensitivity. Quantitatively controlled posthybridization washes combined with an ethidium bromide-prestaining RNA procedure to directly visualize prestained RNA bands at any time during electrophoresis or immediately after electrophoresis, which made the progress of the Northern procedure to be monitored and evaluated step by step, thereby making the experiment reliable and controllable. We also report tips used in the modified Northern protocol, including the moderate concentration of formaldehyde in the gel, the accessory capillary setup, and the staining jar placed into an enamel square tray with a lid used for hybridization. Using our modified Northern protocol, eight rounds of rehybridization could be performed on a single blot. The modification made and tips used ensured the efficient proceeding of the experiment and the resulting good performance, but without using special reagents or equipment.

**Conclusions:**

The modified Northern protocol improved detection sensitivity and made the experiment easy, less expensive, reliable, and controllable, and can be employed in a routine molecular biology laboratory to detect low-expressed mRNAs with heterologous DNA probes in total RNA.

**Supplementary Information:**

The online version contains supplementary material available at 10.1186/s12864-021-08275-w.

## Background

The study of gene expression can provide us with knowledge about gene function and regulation. The multitiered technological methods used for identification of gene expression patterns can be divided into three categories: techniques for detecting the expression of one or a small number of genes, whole transcriptome-based approaches [1], and targeted next generation sequencing assays for the selected specific sets of genes or genomic regions [2]. Northern blot analysis, ribonuclease protection assay, and real-time polymerase chain reaction (qPCR) are the three most commonly used techniques for studying the expression of one or a small number of genes [1, 3, 4]. Among these three methods, qPCR is an often used technology in current molecular biology laboratories due to its extreme detection sensitivity, high specificity and throughput [1, 3, 5, 6]. However, its prominent advantage of higher detection sensitivity also condemns the risk of the publication of inconsistent, irrelevant, and even misleading data based mostly on flawed qPCR results and associated interpretation because of the difference in the quality of RNA samples and the efficiency of reverse transcription (RT), and the inappropriate methodologies selected for the normalization and quantification of data [7, 8].

Compared to qPCR, the major limitations of Northern blot analysis are low detection sensitivity and easy RNA degradation by contaminated exogenous ribonucleases (RNases) in the course of extensive handling of RNA prior to blotting. Furthermore, Northern blotting is a relatively labor-intensive technique because the procedure consists of several steps [1, 3, 4, 9]. Therefore, Northern blotting is not a desirable method for RNA analysis. However, it has the unique advantage of providing information about the expression level and size of the transcript so that it can be used to detect RNA degradation and alternative splice product of the same gene or repetitive sequence motifs, to reveal deletions or errors in transcript processing, and to isolate novel transcripts from heterogeneous mRNA pools [1, 3–5, 9, 10]. In recent years, it has been aptly used to validate and study the size and relative abundance of small noncoding RNAs [11, 12]. Furthermore, because the signal strength obtained from Northern blotting is directly related to the gene copy number in the original sample, rather than being an extrapolated value influenced by the efficiency of RT or amplification as in RT-PCR, the true quantitation of the signal can be obtained by directly comparing changes in the RNA level between samples on a single membrane [9]. Therefore, Northern blotting is arguably a technique that provides highly valid gene expression data [13], and is often used as a gold standard for the validation of data obtained from high-throughput gene expression analysis [9, 13]. In this context, it is necessary to optimize the traditional Northern protocol to make the technique more applicable for standard use.

The basic steps of Northern analysis have remained unchanged since the establishment of this method, but there are alternatives at every step because many variations and improvements have been made to the original protocol [4, 10], such as the modified Northern protocols for specific RNA detection, including the blue native Northern blotting for the detection of RNA in ribonucleoprotein complexes [14], the immuno-Northern blotting for the detection of modified RNA using gel separation and antibodies to modified nucleosides [15], and a recently reported modified Northern blotting, and a modified non-classical variation of Northern blotting, liquid hybridization assay, for sensitive mRNA detection [16]. But perhaps the most important advance in nucleic acid hybridization technology is the use of nonradioactively labeled probes, such as digoxigenin and biotin-based non-isotopically labeled probes [4, 10], and recently reported near infrared fluroscent dye-labeled probes [17]. Nonradioactive probes offer several advantages over radioactive probes, such as improved safety, higher stability, and lower cost [4, 10, 11], but in practice, nonradioactive probes are not widely used in Northern blot analysis, which is most likely a reflection of their actually relatively low sensitivity and signal-to-noise ratio [4, 10, 11]. For an inexperienced investigator, a lot of efforts should be made to optimize the method so that it is reproducible for the particular task at hand. Therefore, this study modifies the Northern protocol using isotope-labeled probes due to the easily reproducible characteristics of the radioactive detection method. Our modifications made and tips used made it easy for the traditional Northern protocol to detect and quantify mRNAs: Posthybridization washes were performed only under moderately stringent conditions, and were quantitatively controlled to maximally retain the specific hybridized probes on the filter, thus improving detection sensitivity; in combination modified washes with a procedure of direct visualization of RNAs in the gel or blotted onto the membrane by ethidium bromide (EtBr)–prestaining RNA before electrophoresis [18], the progress of the whole Northern blotting procedure from the beginning of size-separation to the end of posthybridization detection could be monitored and evaluated step by step, thereby having a reliable and controllable experiment. The study also reported on the tips used to ensure the efficient proceeding of the experiment, including a moderate concentration of 12% formaldehyde in agarose gels to provide adequate capacity to maintain the denatured state of RNAs and, in the meantime, denaturing contaminated exogenous RNases, without compromising the subsequent blotting; measures taken to ensure efficient and successful blotting of size-separated RNAs onto the filter membrane by capillary transfer; a staining jar placed into an enamel square tray with a lid rather than special equipment used for hybridization to protect investigators from radioactivity, however, they still ensured the efficient hybridization of the fixed RNAs on the filter to probes. Using our modified Northern blot procedure, the filter could undergo up to eight rehybridizations cycles. Overall, our modified Northern protocol will not only improve the detection sensitivity, but will also make the experiment of Northern blotting easy, less expensive, reliable, and controllable.

## Results and discussion

### Improved detection sensitivity by modification of posthybridization washes: quantitatively controlled moderate-stringency washes

**Fig. 1:**
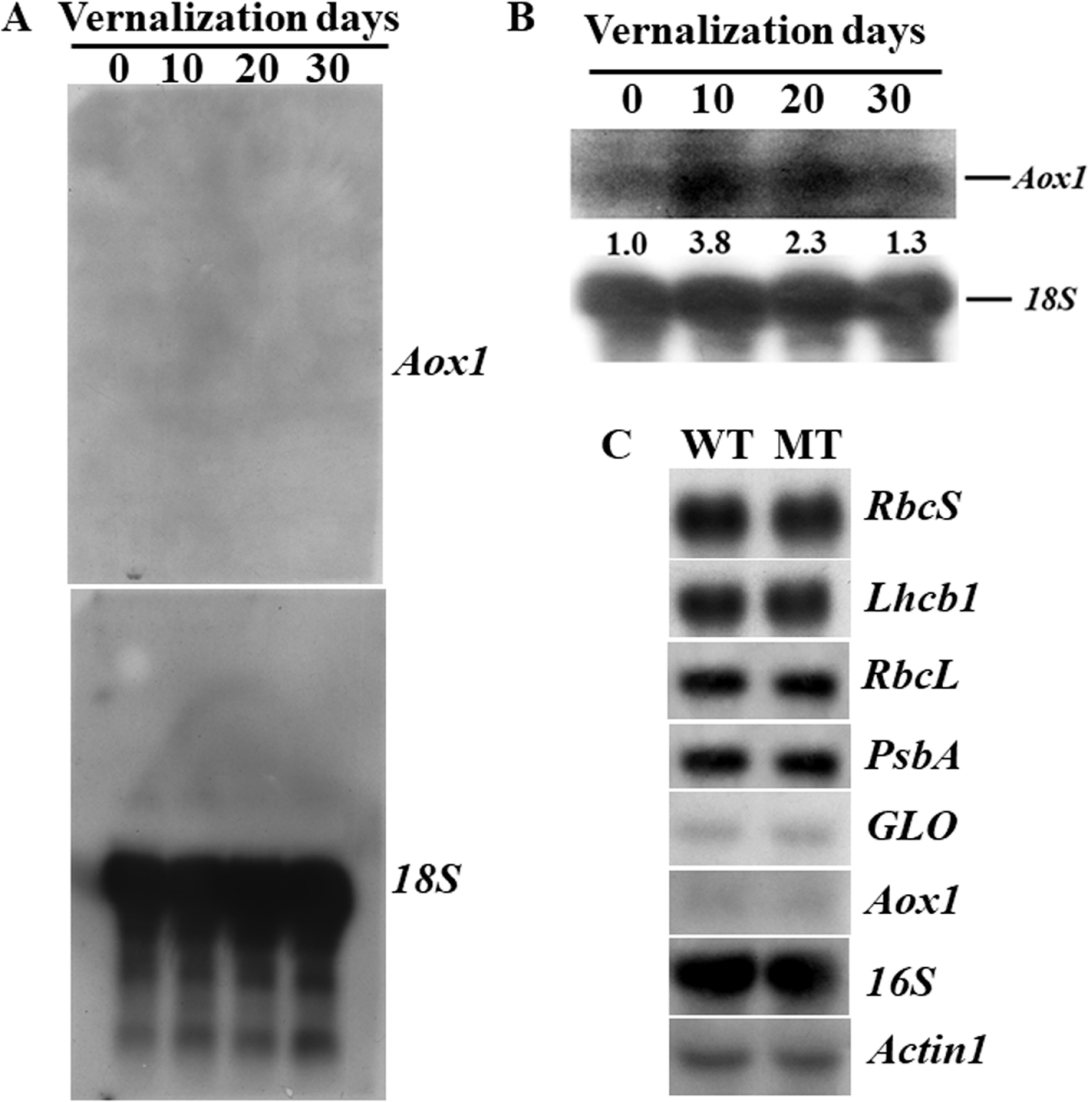
Effects of the modified Northern protocol on the performance of Northern blot analysis. **a** Germinating wheat was vernalized at 0~2 ºC for 0, 10, 20, and 30 d, respectively. The total RNA was isolated and an equal amount of total RNA (50 µg) was resolved using the formaldehyde-agarose gel containing 12% formaldehyde. Gel treatment, transfer of separated RNAs to a positively charged nylon membrane, fixation of transferred RNAs on the membrane, and prehybridization and hybridization were performed, as described in Methods. The traditional posthybridization washes under high and low stringency sequentially for scheduled time were performed according to Clark [20]. The probe used for detection was an *Aox1* cDNA from *N. tabacum*. Hybridization with *18 S* was used as an internal control. The exposure times for the detecting *Aox1* and *18 S* were 5 d and 30 min, respectively. **b** An equal amount of total RNA (20 µg) from vernalized germinating wheat at 0~2 ºC for 0, 10, 20, and 30 d, respectively, was loaded to analyze the level of *Aox1* transcripts. Quantitatively controlled moderate-stringency washes were performed, as described in Materials and methods. The exposure times for the detection of *Aox1* and *18 S* were 2 d and 30 min, respectively. The values below the blot denote the fold-change relative to the germinating wheat without vernalization (0 d), standardized to the *18 S* rRNA content. This experiment was performed twice with similar results. **c** An equal amount of total RNA (20 µg) from the leaves of a chlorophyll reduced mutant of *B. napus* (MT) and its wild type (WT) grown in the field at two-leaf stage was subjected to RNA gel blot analysis following the modified protocol, as described in Materials and methods. Eight different probes were used to rehybridize to the same blot. The experiment performed twice, and the quantification of the hybridization signals from the autoradiographs showed that there was no significant difference in the expression of these genes investigated between the mutant and the wildtype oilseed rape

The principle of detecting specific mRNA in heterogenous mRNA pools by Northern blot analysis is based on the ability of the complementary single-stranded nucleic acid probe to form hybrid molecules with the target, in which after removal of the unbound and nonspecific bound probes, the specific bound probes on the membrane are detected by an appropriate detection technique [4, 10]. Therefore, how to maximally retain the specific bound probes on the filter under the premise of the lower background by removing nonspecific bound probes at the step of posthybridization washes is one of the keys to improve detection sensitivity. However, according to traditional protocols, posthybridization washes are first performed under low stringent conditions to remove the hybridization solution and unhybridized probes, and then performed under high stringent conditions to remove partially hybridized nonspecific bound probes, and each round of washing is performed for at least 10 min [4, 9, 10, 19, 20]. Such prolonged washing can cause nonspecific and specific bound probes to be washdown from the membrane, which may be worse when detecting the expression of low expression genes. One of such examples is the detection of the expression of *Aox1*, a gene expressed in low-copy number that encodes the mitochondrial alternative oxidase (AOX), which is a regulatory component embedded in the inner face of the mitochondrial inner membrane [21]. Successful detection of *Aox1* transcripts by Northern blot analysis can be achieved in two ways: using larger amounts of total RNA (30 µg, e.g. [22]; or even up to 50 µg, e.g. [23]) or poly (A)^+^ RNA (e.g. [24–26]) for analysis, or still using total RNA even in 20 µg of routine use, but changing the condition of posthybridization washes, such as washes only under high stringency, as in Borecký et al. [27]. It should be noted that all of the above examples use homologous probes for analysis. In the case of heterologous probes, due to the lower stability of the hybrids between the target and the mismatched probes, the radioactivity on the filter may be quickly washed down [20]. Figure 1a shows the failure to detect the expression of *Aox1* in vernalized germinating wheat using a probe of *Aox1* cDNA from tobacco [28] in strict accordance with the traditional posthybridization washing procedure [20], even the amount of RNA loaded for analysis was up to 50 µg and the exposure time was extended to 5 d, while detection of *18 S* rRNA on the same filter gave a much stronger signal only after 30 min exposure. In wheat, there are two non-homologous genes (*Waox1a* and *Waox1c*) encoding AOX, and both of them have low copy numbers [26]. The similarity of the nucleotide acid sequences between tobacco *Aox1* cDNA and the two wheat *Aox1* genes (*Waox1a* and *Waox1c*) was 74% and 71%, respectively. Washes under only high stringency, as in Borecký et al. [27], also resulted in the quick washdown of the radioactivity from the filter (data not shown), which may be due to the quick stripping of heterologous probes by strong stringent washes [20]. Our modification was the tradeoff between procedures of the traditional protocol and under only high stringency of Borecký et al. [27], and only under moderate stringency in 1 × SSC/0.1% SDS at 55 ºC, and the indication of the appropriate wash time was the radioactivity level monitored on the membrane until it decreased to 20~50 cps by washing. Moderate-stringency washing can ensure that probes bound specifically are retained on the filter, and those bound nonspecifically are removed, thereby avoiding background signals. It was suggested that the filter membrane showing a Geiger counter reading of 10~20 counts per second (cps) was most suitable for subsequent developing [20]. Our standard was higher because the filter membrane retained higher radioactivity could be re-washed after seeing the results on the autoradiograph. However, once the filter membrane is excessively washed, it cannot be remedied, and the only way is to strip the probe and re-hybridize to the same probe.

Since specific probes were maximally retained on the filter, while background signals were avoided according to our modification, the low expression genes could be easily detected. Figure 1b shows the successful detection of *Aox1* transcripts in 20 µg of total RNA isolated from vernalized wheat by still using the tobacco *Aox1* cDNA [28] as a probe (for full blots, see Suppl. Fig. 1). The signal could be detected after a two-day exposure, suggesting that our modification of posthybridization washes under only moderate stringency improves the detection sensitivity, which can be further verified by the successful detection of *Aox1* transcripts in leaves at two-leaf stage of a chlorophyll-reduced oilseed rape and its wild type still using the same heterologous probe with the same Northern protocol (Fig. 1c) (for full blots, see Suppl. Fig. 2).

Except for the major advantages mentioned in the introduction, one minor advantage of Northern blot analysis is that sequences with only partial homology can be used as hybridization probes [9], which is important because homologous DNA or RNA probes are not always available. However, it can be seen from our results that partially matched heterologous DNA probes can be quickly stripped from DNA-RNA hybrids by high stringency posthybridization washes when the method is applied to monitor the expression of low-abundance mRNAs in total RNA. Consequently, the traditional Northern protocol should be modified for the particular task at hand. Our quantitatively controlled posthybridization washes under only moderate stringency improved detection sensitivity, thus allowing the method to be employed by using heterologous DNA probes to measure the steady state levels of minor mRNA species in total RNA. Therefore, this method is still feasible to detect the expression of low-abundance mRNAs when only heterologous DNA probes are at hand and/or the cell or tissue source is limited because there is no need to isolate poly(A)^+^ mRNAs from a large amount of total RNA.

The disadvantage of our modified Northern protocol is the high background when detecting the expression of low-abundance mRNAs with heterologous DNA probes. This is because posthybridization washes is only performed under moderate stringency, and with a short time in order to avoid quick stripping of heterologous probes from the membrane. For example, our detection of *Aox1* expression in vernalized wheat shoots and in a chlorophyll-reduced oilseed rape mutant at the two-leaf stage showed that the radioactivity on the membrane decreased to about 30 cps only after 11 and 12 min of washing, respectively (Suppl. Table 1). Furthermore, the probes were used directly after labelling for hybridization without purification to remove unincorporated [α-^32^P] dCTP. As a result, unhybridized and nonspecific probes were not adequately stripped. However, the disadvantage of a high background was clearly outweighed by the advantage of obtaining the reproducible experimental results in a convenient and easy way when there was a limitation of the cell or tissue source for RNA gel blot analysis and only heterologous DNA probes were available. The high background in this situation can be lowered using purified labeled probes and/or using homologous probes and better antisense-RNA probes [4, 9] because of the higher stability of the hybrids and the greatest stability of RNA-RNA hybrids, thus the membrane can withstand longer time of posthybridization washes to strip off unhybridized and nonspecific probes.

### Monitoring the progress of Northern blotting: combination procedures of quantitatively controlled posthybridization washes with EtBr-prestaining RNA

The Northern procedure is straightforward, thus it provides an opportunity to evaluate progress at various time points. For Northern analysis, the heart is the transfer of electrophoretically separated RNAs from the gel to a solid support for subsequent fixation and hybridization with specific probes. The quality of the size-separation of RNAs through formaldehyde-agarose gels can be evaluated by visualization of EtBr-stained RNA bands. However, the existence of formaldehyde in the gel can result in high background fluorescence if the gel is stained by conventional methods of incorporating EtBr into the entire gel or staining the gel after electrophoresis, which makes it impossible to visualize EtBr-stained RNA bands immediately after electrophoresis. An alternative staining procedure for prestaining RNA with a low concentration of EtBr (≤30 µg/mL) prior to electrophoresis by heating RNA samples in the presence of EtBr before loading the gel makes it possible to directly visualize EtBr-prestained RNA bands by ultraviolet (UV) light irradiation. Therefore, the verification of RNA integrity and evaluation of the quality of the size-separation can be performed at any time during electrophoresis or immediately after electrophoresis, and the transfer efficiency can also be checked immediately after blotting by viewing the membrane or gel directly on a UV transilluminator [18]. However, it has long been impossible to monitor the progress of posthybridization detection because posthybridization washes were performed for the scheduled time [4, 10, 20] and the results can only be evaluated after viewing the results on the autoradiograph. However, in fact, the behavior of decreasing in the levels of radioactivity on the filter during washing can provide information on the efficiency of probe-labeling and hybridization, because the rapid decrease in radioactivity on the filter during washing may be due to poor labeling or insufficient hybridization [4, 20]. Therefore, our quantitatively controlled posthybridization washes enables posthybridization detection to be evaluated in advance during posthybridization washes, rather than after viewing the image on the autoradiograph like the conventional posthybridization washing procedure for the scheduled time. Overall, the combination of procedures of EtBr-prestaining RNA with quantitatively controlled posthybridization washes makes it possible to monitor and evaluate the progress of Northern blotting step by step, thereby making the experiment reliable and controllable.

### Efficient proceeding of Northern blot analysis with less expense: some tips

The RNA samples subjected to Northern blot analysis are size-separated through an agarose-gel under denaturation conditions, and then the gel is treated by sequentially soaking in different solvents for its subsequent transfer [4, 10, 19]. Such extensive handling of RNA prior to blotting placed RNA in the danger of degradation by ubiquitous RNases, which are introduced accidently and subsequently resulted in the severely compromised outcome of Northern blotting. Therefore, an RNase-free environment should be created and maintained during these processes, especially when the isolated RNA samples are usually dissolved in a solvent such as DEPC-H_2_O without denaturants to inactivate RNases. In the process of RNA electrophoresis through formaldehyde-agarose gels, it is unnecessary to include special reagents in the system to maintain an RNase-free environment, because formaldehyde used for denaturing and maintaining the denatured state of RNA is also a denaturant of the RNase through the mechanism of formaldehyde reaction with amino and imino groups of amino acids to form Schiff bases [18]. For this reason, a higher concentration of formaldehyde was preferred to provide adequate denaturation to RNases and to compensate for the loss of formaldehyde through diffusion from the gel into the buffer during electrophoresis [4]. However, the existence of formaldehyde in agarose gels hinders subsequent blotting, thus a prolonged soaking in DEPC-H_2_O or other solvents is required to remove formaldehyde in the gel prior to setting up the transfer [10]. Therefore, the concentration of formaldehyde in the gel should be moderate, which is also a fairly variable parameter in different protocols, such as often used at 18% [4, 19] or 16% [20], or much lower even at 5% [10] or 3% [9]. In our practice, agarose gels containing 12% formaldehyde were found to achieve the best combination of denaturation to both RNA and RNases during electrophoresis, without hindrance to subsequent blotting of size-separated RNAs to the solid support, simply by soaking the gel in DEPC-H_2_O for 10 min.

**Fig. 2:**
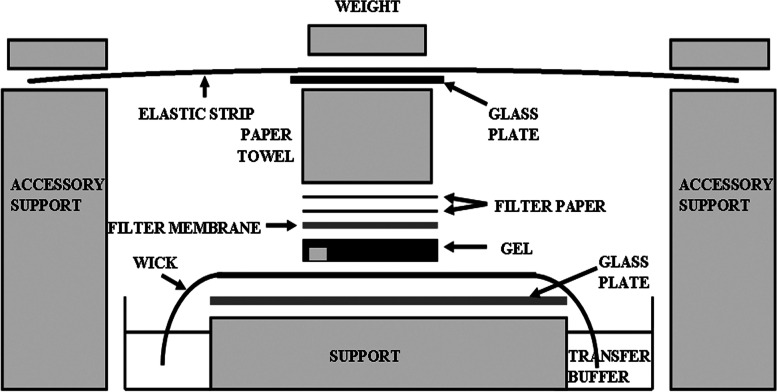
Modified capillary transfer setup for blotting of size-separated RNA to a filter membrane. The transfer setup is the same as described in most protocols. Our modification was to add an accessory setup in accordance with Materials and methods to ensure good contact between each component of the setup, and no shift of the center of the gravity of the top weight as a result of the uneven pressure distribution of the top weight to the paper towels underneath

Electrophoretically resolved RNA can be blotted onto a solid support using a commercially fast transfer apparatus, such as electroblotting and a vaccumblotting apparatus, but the traditional simple and no instrumentation-required capillary transfer can still work well [4, 10]. We used the simple and economical upward capillary transfer at neutral pH, and the transfer setup was basically the same as that described in most protocols [4, 10, 19, 20]. This simple method is quite reliable, but based on our experience, we found that the deformation of the wetted paper towels might result in inefficient transfer because the wetted paper towels would extend horizontally, and then would be deformed and creased. Therefore, the gel over the area of the crease may lead to the non-uniformity of capillary action. In addition, the wetted area of paper towels would shrink vertically. Due to the uneven wetting performance of the paper towels, the vertical shrinkage of paper towels was also uneven, resulting in uneven pressure distribution from the top weight to the paper towels underneath, which in turn causes deviations in the center of gravity of the top weight, and finally the collapse of the setup. To avoid these problems, the first measure was to flatten the first few layers of paper towels placed on the filter paper. However, even if so, the transfer setup easily collapsed during overnight transfer as soon as the pressure of the top weight was unevenly distributed to the paper towels underneath, especially when the gel was narrow if only two or three lanes were used and the unused area of the gel was trimmed away as recommended (e.g. [10]). Our major precaution was to add an accessory setup to both sides of the transfer setup (Fig. 2) to ensure good contact between the various components of the transfer setup, and ensure that the pressure of the top weight is evenly distributed to the paper towels underneath, thus the center of gravity of the top weight does not shift during transfer process. To avoid the diffusion of bands, the top weight should not be too heavy, as a 200 g of weight is sufficient; to ensure the complete transfer, the transfer time was up to 18 h.

**Fig. 3:**
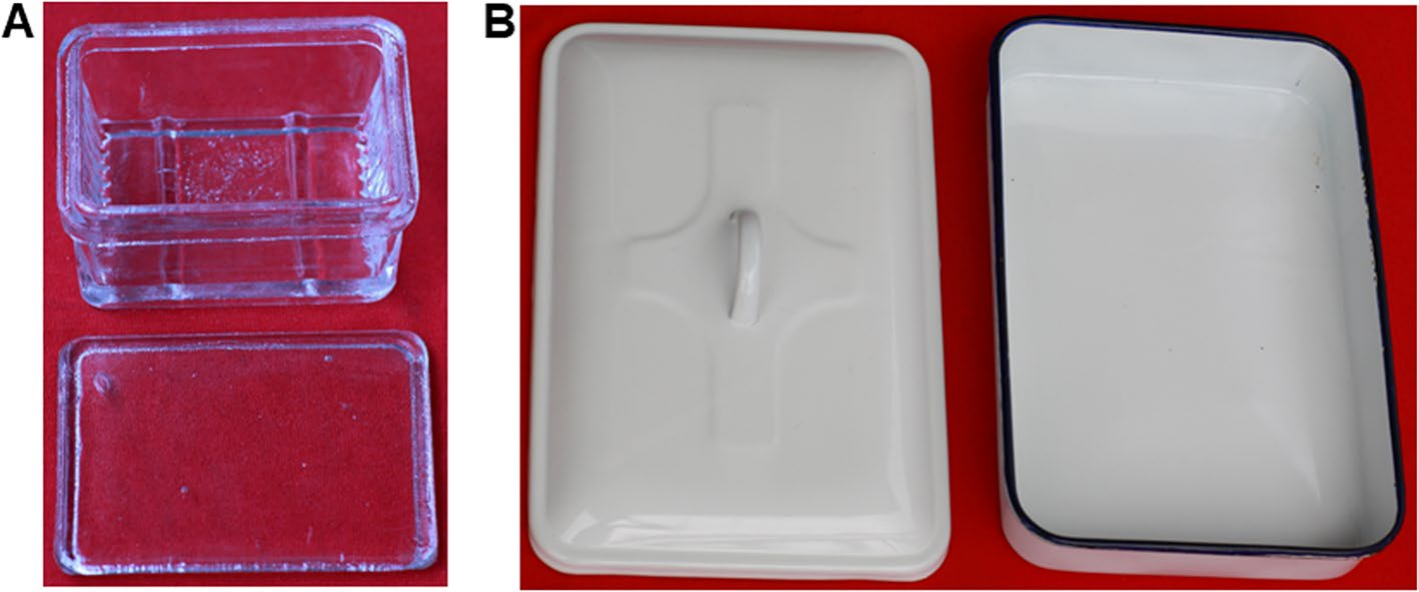
Gadgets used for hybridization. **a** Staining jar. Normally, the staining jar is used for staining tissue slices, where the gadget was used for prehybridization and hybridization, and its bottom size is 7.2 cm × 5.2 cm, with the thickness of its wall being 0.5 cm. The hybridization of the larger blotted membrane was performed in a glass Petri dish; **b** Enamel square tray with lid: During hybridization, the staining jar was placed into an enamel square tray, after being covered with the lid, the enamel tray was taken into a lab incubator to hybridize at 42 ºC. The thick glass of the staining jar, the metallic material and the enamel of the enamel square tray can protect investigators from hazardous radiation, and prevent the evaporation of toxic formamide and the spillage of radioactive materials

To prevent higher doses of radioactivity, the hybridization oven is the best choice for hybridization of Northern blots, whose thick glass tubes can efficiently shield radioactivity. We performed prehybridization in a staining jar (Fig. 3a). During hybridization, the staining jar was placed into an enamel square tray with a lid (Fig. 3b). After covering the lid, the enamel tray was taken into a laboratory incubator and stationary hybridization was allowed at 42 ºC for 15 h. The quadrate bottom of the staining jar made it possible to use small volumes of liquid, thus making the probe more concentrated. Radioactivity could be reduced by the thick glass of the staining jar, and shielded by the metallic material and the enamel of the enamel square tray. Our hybridization system can also prevent evaporation of toxic formamide and spillage of radioactivity. Compared to other recommended economical homemade devices, such as plastic boxes and heat-sealed plastic bags [4, 10, 20], the gadgets we used worked equally well, but had advantages of safety to investigators and simple operation.

Overall, these tips enable experiments to be efficiently carried out but without using special equipment, so the costs of the experiment are greatly reduced, and the experiment can be easily performed and reproduced in a simple equipped molecular biology laboratory even by a novice investigator.

### The best example of successful protocol modification: Eight rounds of rehybridization on a single blot

Table 1 summarizes the modifications made in this study and the resulting performance advantages of Northern blotting. As a whole, modifications made and tips used reported here not only improved detection sensitivity, but also enabled the efficient proceeding of Northern blotting, so the good performance of Northern blotting was achieved. Figure 1c shows the expression of photosynthetic-associated genes in leaves at the two-leaf stage of a chlorophyll-reduced mutant of oilseed rape detected in 20 µg of total RNA using eight different probes. Full blots of these probes are provided in Suppl. Fig. 2, and the wash time to the desired counts and the exposure time for each probe are listed in Suppl. Table 1. Of the eight genes, *GLO* that encodes glycolate oxidase in peroxisome, is also a low expression gene in juvenile leaves [29]. To the best of our knowledge, eight rounds of rehybridization is currently the most reported reprobing time, which can also meet the requirements for the number of reprobing genes in most individual gene expression studies. The results of the current study indicated that the expressions of all eight genes investigated do not have significant difference in the leaves at the two-leaf stage of the mutant and its wildtype oilseed rape (Fig. 1c). Therefore, our successive research used the fully expanded cotyledon as materials because the differences in both gene expression and phenotype were quite obvious [30]. The distinct, sharp smear-free hybridization signal suggests that even if RNAs were extensively handled prior to blotting, RNA degradation did not occur; the clear hybridization signals were detected in all eight rounds of hybridization, indicating an efficient transfer of separated RNAs to the filter membrane and immobilization of the transferred RNAs on the solid support; good performance proved that our hybridization system using small gadgets was equivalently efficient. Compared with the newly reported improved Northern protocol [16], no special reagents or apparatus were used in our protocol, and thus the financial demands for the experiment were greatly reduced. Furthermore, our modifications enabled us to directly and easily check the proceeding of Northern blotting in the steps of electrophoresis, capillary transfer, and posthybridization detection. Therefore, the experiment can be monitored and evaluated step by step. Overall, the modified Northern protocol not only improved detection sensitivity, but also made the Northern blotting experiment easy, less expensive, reliable, and controllable.

**Table 1:** Summary of the modifications made for the conventional Northern blotting technique and advantages resulted

Steps	Conventional Northern blotting	Modifications	Advantages
RNA denaturation before electrophoresis	No EtBr in RNA samples	Heating RNA samples in the presence of EtBr before loading the gel	Possible to verify the integrity of RNA and evaluate the quality of the size-separation at any time even during electrophoresis, to check the transfer efficiency immediately after blotting
Formaldehyde concentration in agarose gels	Great variation in different protocols, much higher (e.g., 18%) or even much lower to 3%	A moderate concentration of 12%	Achieved the best combination of denaturation to both RNA and RNases during electrophoresis and no hindrance to subsequent blotting
Capillary transfer	Only transfer setup	An accessory setup along the both sides of the transfer setup	Avoidance the collapse of the setup and ensured the efficient and successful transfer
Hybridization	In hybridization oven	In a staining jar placed into an enamel square tray with a lid	Greatly decreased cost while still efficiently protection of investigators from radioactivity and proceeding of the hybridization
Post-hybridization washes	Followed by a blocking step of under high and low stringency sequentially for scheduled time	Under only moderate-stringency till the level of radioactivity retained on the filter decreased to 20~50 cps	Maximally retaining the specific hybridized probes on the filter thus improved the detection sensitivity; Possible to monitor the progress of posthybridization detection

## Conclusions

Northern blot analysis has a unique advantage of providing information on expression level and native size of the RNA, and the direct quantitation of the signal also makes it a technique that provides highly valid expression data, so it is still a widely used technique as a gold standard for the direct study of gene expression at the RNA level and to detect transcript sizes [4, 9, 13]. However, the low detection sensitivity, high quality of RNA required, and the long time it takes to complete an analysis make it an undesirable method for analyzing RNA [1, 3, 4, 9]. Our aim was to optimize the traditional protocol by tweaking at the steps of electrophoresis, capillary transfer, hybridization, and posthybridization washes, so as to make the technique more applicable for standard use. Although the steps were not simplified and the time to complete was not saved, the modified Northern protocol improved the detection sensitivity and made the multi-step, labor-intensive experiment easy, less expensive, reliable, and controllable. Therefore, this technique can be easily reproduced in a routine molecular biology laboratory and used to monitor low-abundance mRNA expression with heterologous DNA probes in total RNA, even when the cell or tissue source are limited and homologous DNA or RNA probes are not readily available.

## Methods

### RNA isolation and size-separation through formaldehyde agarose gels

Total RNA was isolated from vernalized shoots of *Triticum aestivum* at 0~2 ºC for 0, 10, 20, and 30 d, respectively, or from leaves of the field-grown chlorophyll reduced mutant in *Brassica napus *[30] and its wild type at two-leaf stage, essentially according to the method of Zhang et al [31] and with modifications described in Zhang et al. [32]. The RNA was dissolved in diethylpyrocarbonate (DEPC)-water and an equal aliquot (20 or 50 µg) was heat-denatured at 65 ºC in the presence of 10 µg/mL EtBr and resolved using the agarose gel containing 12% formaldehyde according to the method of Zhao et al. [18].

### Capillary transfers

After electrophoresis, the gel was soaked in DEPC-H_2_O for 10 min with gentle shaking to remove formaldehyde, and then soaked in 50 mM NaOH for 20 min to partially hydrolyze the RNAs in the gel. After being soaked in 20 × SSC (1 × SSC is 0.15 M NaCl, 0.015 M sodium citrate, pH 7.0) for 45 min, the partially-hydrolyzed RNAs were transferred to a positively charged nylon membrane (Boehringer Mannheim, Germany) using a conventional capillary method for 18 h as described in most protocols [4, 10, 19, 20]. Our modification was to add an accessory setup by placing two supports on both sides of the transfer setup, and just ensuring that the height of the support was just lower than that of the paper towels, and then placing an elastic strip (using a long plastic ruler) across the glass plate and two supports. After pressing both ends of the strip, a 200 g weight was placed in the middle of the strip above the glass plate (Fig. 2). At the beginning of the transfer, when the first several layers of paper towels on the filter paper became wet, they were withdrawn and flattened with the pull of the hands, after which the extended edges were trimmed to match the gel size, the flattened paper towels were returned to the transfer setup, and then the transfer process was resumed. After blotting, the gel was directly placed on an UV transilluminator to check the transfer efficiency; the blotted membrane was briefly rinsed in 2 × SSC, and then placed between two sheets of filter paper and allowed to air dry. After baking at 80 °C for 2 h, the filter was directly subjected to the hybridization step or stored sandwiched between two sheets of filter paper, which was placed in a dry location out of light until further use.

### Prehybridization and hybridization

The blotted membrane was prehybridized and hybridized with ^32^P-labeled probes at 42 °C according to Clark [20]. Prehybridization was performed in a glass staining jar, whose bottom size was 7.2 cm × 5.2 cm and the thickness of the wall was 0.5 cm (Fig. 3a), which was with gentle shaken. After adding radioactively labeled probes to the prehybridization buffer, the staining jar was placed into an enamel square tray with a lid (Fig. 3b). After covering the lid, the enamel tray was taken into a laboratory incubator and stationary hybridization was allowed at 42 ºC for 15 h. The hybridization probes used for the detection of gene expression in vernalized wheat were the *Aox1* cDNA from *Nicotiana tabacum *[28] and a partial fragment of *18 S* rDNA by amplification from *B. napus*, respectively. The hybridization probes used for detection of gene expression in *B. napus* were *Aox1* as described above, *RbcS*, *Lhcb1*, *GLO*, *PsbA* and *Actin 1* as described in Zhang et al. [30], *16 S* rDNA as described in Zhu et al. [33], and *RbcL* as described in Wang et al. [34]. The information of all probes used in this study is listed in Suppl. Table 2. The probe fragments were labeled with 50 µCi of [α-^32^P] dCTP (380 MBq/mL) by the random primer method using a Random Primer DNA Kit Ver 2.0 (TaKaRa Bio Inc., Dalian, China) according to the manufacturer’s instructions. The labeled probes were used directly for hybridization without purification to remove unincorporated nucleotides. To obtain DNA probes with high specific activity, the length of the template DNA for labeling should be greater than 300 bp, and the amount should be lower approximately 25 ng. The specific activity of the labeled probes was 1~1.6 × 10^9^ dpm/µg.

### Posthybridization washes

Posthybridization washes were performed under only moderately stringent conditions in 1 × SSC/0.1% SDS at 55 ºC. A Geiger counter was used to monitor the level of radioactivity on the membrane until it was reduced to 20 to 50 cps. The wash solution was changed every 10 min whenever the level of the radioactivity on the membrane was too high. The membrane washed to the desired counts was placed between two sheets of filter paper to blot excessive buffer, and then wrapped in plastic wrap and exposed to an X-ray film with intensifying screens at -80 °C. Hybridization signals from the autoradiographs were quantified using the analysis tool of the SynGene bioimaging system. Posthybridization washes were also performed as the traditional protocol under low and high stringency sequentially for scheduled time (in 2 × SSC/0.5% SDS at room temperature twice for 10 min, and in 0.5 × SSC/0.2% SDS at 62 ºC for 10 min, respectively) according to Clark [20], or under only high stringency in 0.5 × SSC/0.2% SDS at 62 ºC according to Borecký et al. [27].

### Stripping the probe

After recording the image, the probe on the damp membrane was stripped in 0.1 × SSC/0.1% SDS (preheated to 100 ºC), and then reprobed with another probe, as described by Zhao et al. [18]. The membrane could be immediately reused or stored as described above. It should be noted that the filter must not dry out, otherwise, the bound hybridized probe would be difficult to remove.

Theoretically, the blotted membrane can withstand rehybridization many times, but after each cycle of hybridization and probe stripping, a fraction of RNA immobilized on the membrane leaches away, so the signal strength decreases progressively with each use [4]. For this reason, the blotted membrane can only be stripped and rehybridized for limited times, and the reprobing was recommended to follow the order of expected low to high abundance signals.

## Supplementary Information

**Additional file 1.**

## Data Availability

All data generated or analyzed during this study are included in this published article (and its supplementary information files).

## Abbreviations

cps: Counts per second
DEPC: Diethylpyrocarbonate
EtBr: Ethidium bromide
PCR: Polymerase chain reaction
qPCR: Real-time PCR
RNase: Ribonuclease
RT: Reverse transcription
UV: Ultraviolet
