# Supplementary material for: Modified Northern blot protocol for easy detection of mRNAs in total RNA using radiolabeled probes
Source: BMC Genomics. 2022 Jan 20;23:66. doi: 10.1186/s12864-021-08275-w (PMC8772191; doi:10.1186/s12864-021-08275-w)

**Suppl. Table 1. The wash time to the desired counts and the exposure time for each of 8 probes in Fig. 1C.**

| Gene name | Probe type | Expression level | Wash time to the desired counts (min) | Exposure time (h) |
|---|---|---|---|---|
| *16S* | Homologous DNA | High | 48 | 3 |
| *Actin1* | Homologous DNA | Moderate | 16 | 14 |
| *Aox1* | Heterologous DNA | Low | 12 | 48 |
| *GLO* | Homologous DNA | Low | 14 | 24 |
| *Lhcb* | Heterologous DNA | High | 16 | 6 |
| *psbA* | Homologous DNA | High | 52 | 3 |
| *RbcS* | Homologous DNA | High | 32 | 3 |
| *RbcL* | Heterologous DNA | High | 18 | 5 |

An equal amount of total RNA (20 μg) from the leaves of a chlorophyll reduced mutant of *B. napus* and its wild type grown in the field at two-leaf stage was subjected to RNA gel blot analysis. After hybridization, the blot was washed under only with moderate-stringency until the level of radioactivity retained on the filter decreased to 20~50 counts per second. After that, the blot was exposed to an X-ray film with intensifying screens at -80 °C. After recording the image, the probe on the membrane was stripped, and then reprobed with another probe. Eight different probes were used to rehybridize to the same blot.

**Suppl. Table 2. The information of probes used in this study.**

| Probe | Probe source | Probe length | Primers used for amplification |
| --- | --- | --- | --- |
| *16S* | a *Bam*H I insert of *Zea mays Bam*H I digested plastid genome at the site of Bam 17` in plasmid pBR322 | 2.5 kb | N/A |
| *18S* | a partial fragment of *Brassica napus* 18S rDNA | 678 bp | Forward: 5`-GCC TAC GCT CTG GAT ACA TTA-3`<br>Reverse: 5`-AGA ACA TCT AAG GGC ATC ACA-3` |
| *Actin1* | a partial coding sequence for *Brassica napus* Actin1 | 505 bp | Forward: 5`-GTG ACA ATG GAA CTG GAA TGG-3<br>Reverse: 5`-AGA CGG AGG ATA GCG TGA GG-3 |
| *Aox1* | an *Eco*R I-*Hin*d III insert of the cDNA for *Nicotiana tabacum* alternative oxidase in plasmid pBR322 | 1.1 kb | N/A |
| *GLO* | a full coding sequence for *Brassica napus* glycolate oxidase | 1 104 bp | Forward: 5`-ATG GAG ATC ACT AAC GTT ACC-3`<br>Reverse: 5`-GTA TAA CCT GGG CAA ATG GCG-3` |
| *Lhcb1* | a *Pst* I insert of the cDNA for *Hordezim vulgare* for light-harvesting chlorophyll *a*/*b* protein in plasmid pBR322 | 850 bp | N/A |

| | | | |
|---|---|---|---|
| *psbA* | a full coding sequence for *Brassica napus* PSII reaction centre D1 | 1 062 bp | Forward: 5`-ATG ACT GCA ATT TTA GAG AGA CGC-3`<br>Reverse: 5`-TTA TCC ATT TAT AGA TGG AGC CTC AAC-3` |
| *RbcS* | a full coding sequence for *Brassica napus* Rubisco small subunit precursor | 546 bp | Forward: 5`-ATG GCT TAC TCT ATG CTC TCC TC-3`<br>Reverse: 5`-TTA AGC ACC GGT GAA GCT TGG-3` |
| *RbcL* | a partial coding sequence for *Ginkgo biloba* Rubisco large subunit | 616 bp | Forward: 5`-GTT TGG ACC GAT GGA CTT AC-3`<br>Reverse: 5`-AAC CTC CCG TCA GAT AGT CA-3` |

**Suppl. Figure 1. The full blots of the detection of the expressions of *Aox1* and *18S* in Fig. 1A.** An equal amount of total RNA (20 μg) from vernalized germinating wheat at 0~2 ºC for 0, 10, 20, and 30 d, respectively, was loaded to analyze the level of *Aox1* transcripts. Quantitatively controlled moderate-stringency washes were performed, as described in Materials and methods. The exposure times for the detection of *Aox1* and *18S* were 2 d and 30 min, respectively.

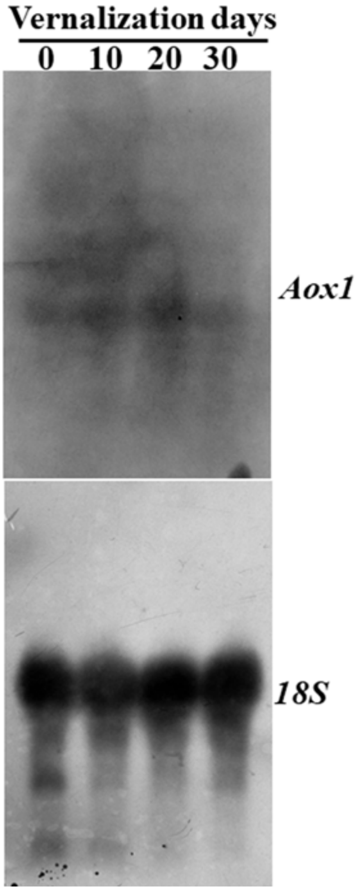

**Suppl. Figure 2. The full blots of the detection of the expressions of eight genes in Fig. 1C.** An equal amount of total RNA (20 μg) from the leaves of a chlorophyll reduced mutant of *B. napus* (MT) and its wild type (WT) grown in the field at two-leaf stage was subjected to RNA gel blot analysis following the modified protocol, as described in Materials and methods. Eight different probes were used to rehybridize to the same blot. The approximate transcript sizes were as follows: 1.5 kb of *16S*, 1.5 kb of *Actin1*, 1.4 kb of *Aox1*, 1.5 kb of *GLO*, 1.2 kb of *Lhcb*, 1.2 kb of *psbA*, o.7 kb of *RbcS*, and 1.6 kb of *RbcL*.

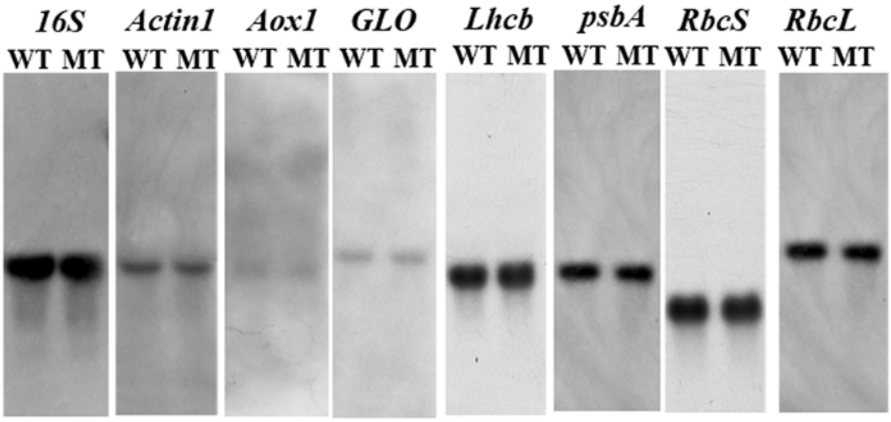

Supplement: Supplementary file 1 — Additional file 1. [file 12864_2021_8275_MOESM1_ESM.pdf]
